# Pointing and pantomime in wild apes? Female bonobos use referential and iconic gestures to request genito-genital rubbing

**DOI:** 10.1038/srep13999

**Published:** 2015-09-11

**Authors:** Pamela Heidi Douglas, Liza R. Moscovice

**Affiliations:** 1 Department of Primatology, Max Planck Institute for Evolutionary Anthropology, Deutscher Platz 6, D‐04103 Leipzig, Germany

## Abstract

Referential and iconic gesturing provide a means to flexibly and intentionally share information about specific entities, locations, or goals. The extent to which nonhuman primates use such gestures is therefore of special interest for understanding the evolution of human language. Here, we describe novel observations of wild female bonobos (*Pan paniscus*) using referential and potentially iconic gestures to initiate genito-genital (GG) rubbing, which serves important functions in reducing social tension and facilitating cooperation. We collected data from a habituated community of bonobos at Luikotale, DRC, and analysed n = 138 independent gesture bouts made by n = 11 females. Gestures were coded in real time or from video. In addition to meeting the criteria for intentionality, in form and function these gestures resemble pointing and pantomime–two hallmarks of human communication–in the ways in which they indicated the relevant body part or action involved in the goal of GG rubbing. Moreover, the gestures led to GG rubbing in 83.3% of gesture bouts, which in turn increased tolerance in feeding contexts between the participants. We discuss how biologically relevant contexts in which individuals are motivated to cooperate may facilitate the emergence of language precursors to enhance communication in wild apes.

While there is debate concerning the time frame in hominid evolutionary history when language-like communication emerged^1^,^2^, there is general agreement that many of the neural structures and cognitive capacities underlying modern human language have phylogenetically more ancient roots^3^,^4^. Such pre-adaptations likely included socio-cognitive abilities that facilitated joint attention and theory of mind^5^, and the use of symbolic signals to refer to external objects, actions, or goals^6^. The latter could be achieved either through imitation of action sequences to represent desired outcomes^7^, or by linking arbitrary sounds or gestures to specific communicative goals^8^. There is evidence that nonhuman primates also possess some of these language precursors^3^,^9^, although this is a topic of ongoing debate^10^,^11^.

The ability of nonhuman primates to use referential manual or bodily gestures in symbolic signalling is especially relevant for exploring the evolutionary origins of language, since primates appear to have more flexibility and control over their gestural than vocal signals^8^. Among various forms of referential gestures, pointing is of special interest, due to its role in facilitating joint attention by visually orienting the recipient to a referent in the immediate environment which may then become a focus of shared attention^12^,^13^,^14^. By enhancing triadic communication between individuals, the ability to point has been linked to facilitating cooperation and behavioural coordination in humans^15^. Iconic gesturing is another important type of symbolic communication, in which signallers create a visual representation of some aspect of an object, action, or event^7^,^16^,^17^. Pantomime is a type of iconic gesturing in which the referent or goal is intentionally re-enacted, typically resulting in unambiguous communication^18^,^19^.

Humans use pointing and pantomime to communicate flexibly about objects and events either immediately present or distant in time or space, and with others who are motivated to understand their communicative intentions. The ability to use referential communication within a shared attentional framework has been identified as critical in the transition from ape gestures to the emergence of uniquely human cooperative communication^15^. The capacity of nonhuman primates to communicate using pointing or iconic gestures has been documented in captivity; however, these studies typically involved either a small subset of signallers interacting with human experimenters^19^,^20^, or a setting where regular contact with humans makes it difficult to rule out the influence of enculturation on the acquisition of the gestures^21^. The few examples of referential or potentially iconic communication among primates in the wild consist of anecdotal or rare events (bonobos (*Pan paniscus*):^22^; chimpanzees (*Pan troglodytes*):^23^,^24^,^25^). Interestingly, there are larger data sets from other taxa in natural settings that meet current criteria for referential gesturing (fish^26^; corvids:^27^), but comparable observations have not yet been documented in wild primates.

Here we describe two novel gestures used by wild female bonobos: a form of pointing^28^,^29^, termed foot-pointing, through which females refer to their sexual swellings, and a form of pantomime^30^, termed hip shimmy, that incorporates the action involved in genito-genital rubbing and is thus potentially iconic. Neither form of gesture was previously known to occur among nonhuman primates in natural environments^30^,^31^. We present evidence that both of these gestures serve important functions in the context of female socio-sexual behaviour. Among female bonobos, socio-sexual behaviour consists primarily of genito-genital rubbing (hereafter GG rubbing) in which two females mount and embrace each other in a ventro-ventral position and use rapid lateral hip movements to rub their genital swellings together^32^. GG rubbing in bonobos differs from female-female genital contacts that are occasionally observed in other primates, both in the frequency of its occurrence and in the important functions to which it has been linked. These functions include facilitating the integration of newly immigrant females into social groups^33^, increasing social standing^34^, regulating social tension^35^, and promoting cooperation^36^ especially in feeding contexts. Given the varied functions of GG rubbing, innovations that enhance a signaller’s ability to request GG rubbing, or that influence the likelihood that receivers will coordinate their behaviour with the signaller, may have important fitness implications for female bonobos.

## Results

We observed n = 169 independent bouts of referential or potentially iconic gestures, involving n = 152 foot-pointing gestures and n = 43 hip shimmy gestures. Gesture bouts consisted of a single foot-pointing gesture (60.4%, n = 102), a single hip shimmy (11.8%, n = 20), or at least one foot-point or hip shimmy performed in succession or in sequences with other gestures (27.8%, n = 47). In all but two cases, gesture bouts occurred in social contexts, in which at least one other individual was within visual range of the signaller.

Of the fourteen mature, long-term resident females who were observed during 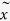 = 1080 hours per female (range: 494–1430), eleven were both signallers and recipients of foot-pointing or hip shimmies. The other three females were recipients, but were not observed to produce these gestures. An additional nine females–five temporary female residents and four juveniles who were all offspring of gesture producers–were recipients but not signallers (Tables 1 and 2). We observed high inter-individual variation in the use of foot-pointing and hip shimmy gestures. Only a subset of signallers produced the hip shimmy gesture, with 79.4% of hip shimmies produced by one female. In comparison, all but one signaller produced the foot-pointing gesture, although some females used this gesture more frequently than others. Despite this variation, there was no relationship between a female’s frequency of gesturing and her total observation time (Spearman, n = 14, r = −0.189, *P* = 0.517).

Of the n = 138 analysed gesture bouts, nearly all foot-pointing (98.1%, n = 103) and hip shimmies (96.9%, n = 32) were observed in feeding contexts. Most gestures occurred while individuals fed on non-monopolisable fruits (79.3%, n = 107), or on highly preferred, monopolisable foods (fruits of *Annonidium* or *Treculia* species, or meat, 18.5%, n = 25). In addition, 31.2% (n = 43) of gesture bouts occurred following a change in social context, due to one or both of the participants entering a new feeding site or reuniting after a fusion of two parties.

### Evaluating criteria for intentionality, referentiality, and iconicity

#### Directed at a recipient

Signallers performed the majority of foot-pointing gestures (94.3%, n = 99) and hip shimmies (97.0%, n = 32) when within close proximity (2 m) to the recipient, or immediately prior to moving within close proximity to the recipient.

#### Adjusted to the attentional state of the recipient

In 100% (n = 33) of hip shimmy bouts and 92.4% (n = 97) of pointing bouts, gestures were performed when the recipient was fully attending the signaller, i.e., facing 0–45°. In the remaining pointing bouts, the recipient was within the visual range of the signaller, but her head was oriented 45–90° (n = 5), or 90–180° (n = 3) away from the signaller when the first foot-pointing gesture was performed.

#### Mechanically ineffective

Females who used foot-pointing gestures made contact with their swellings during 32.3% (range: 0–85.7%) of their gesture bouts. Whether the signaller touched her swelling during a pointing gesture had no influence on the likelihood that she would engage in GG rubbing with the recipient following the gesture (likelihood ratio test: χ^2^ = 1.245, df = 1, *P* = 0.264). In only one unsuccessful gesture bout, the signaller incorporated a brief touch to the recipient’s swelling as a form of communicative persistence, but this touch did not accomplish the goal of GG rubbing and therefore would not be considered mechanically effective.

#### Incorporated a relevant referent or action related to the goal

All gestures incorporated either the relevant referent (the signaller’s sexual swelling, n = 105) or action (vigorous lateral hip movement, n = 33) involved in the dominant response following the solicitation, namely GG rubbing with the recipient of the gesture.

#### Goal-directed

**Figure 1:**
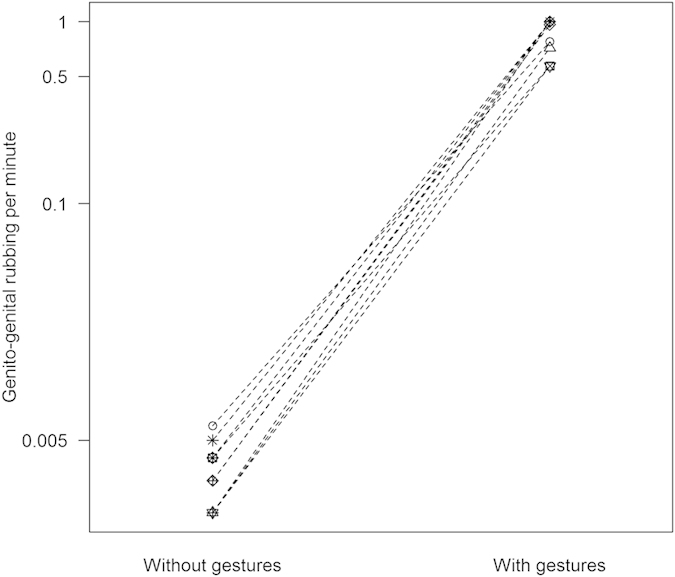
Rates of GG rubbing without and with referential or iconic gestures. In social feeding contexts involving non-monopolisable foods, rates of GG rubbing increased following foot-pointing and hip shimmies (Wilcoxon: T^+^ = 55, *P* = 0.002). Symbols represent n = 10 females. The data are presented on a log-transformed y-axis to better visualise the variation in GG rubbing rates.

Almost all pointing bouts (80.0%, n = 84) and hip shimmy bouts (93.9%, n = 31) resulted in GG rubbing between the signaller and the recipient immediately following the bout. At the individual level, in the subset of social feeding contexts involving non-monopolisable foods, rates of GG rubbing were significantly higher following foot-pointing or hip shimmy gestures compared to instances when females did not use these gestures (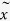 = 0.982 (range: 0.571–1) vs. 0.004 (range: 0.002–0.006) GG rubbing events per minute, Wilcoxon signed-ranks test: T^+^ = 55, n = 10, *P* = 0.002; Fig. 1 and Supplementary Table 2).

#### Communicative persistence

**Figure 2:**
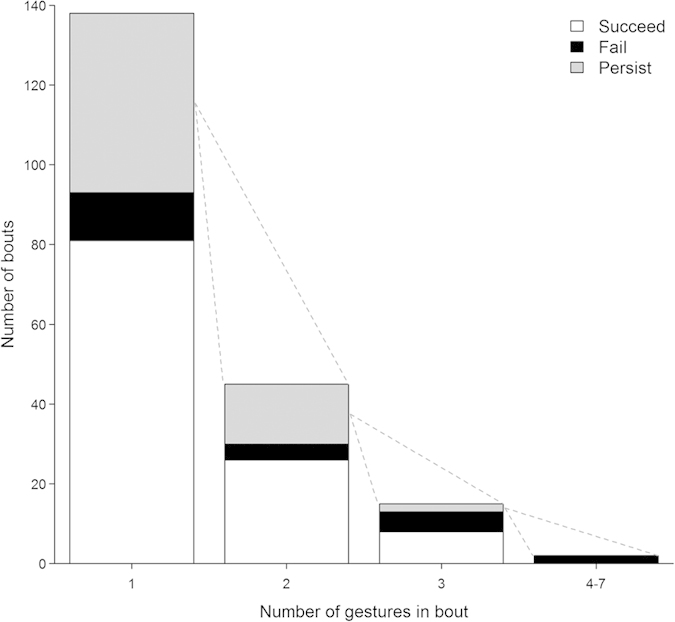
Frequencies and outcomes of gesture bouts without and with persistence. Data are presented for n = 138 independent gesture bouts. Shading indicates the outcome following the last gesture in the bout. Succeed = GG rubbing; fail = no GG rubbing and no persistence; persist = initiation of another gesture within the same bout.

The majority of bouts (67.4%, n = 93) involved a single foot-pointing or hip shimmy gesture. Of these, 85.1% (n = 63) of foot-pointing and 94.7% (n = 18) of hip shimmies resulted in GG rubbing (Fig. 2). On only three occasions did a female initiate a second gesture bout directed towards the same recipient immediately following a positive response, and in all three cases the signaller and recipient had another GG rubbing interaction. When single gestures did not lead to GG rubbing, signallers showed persistence in 77.5% (n = 31) of pointing attempts and 87.5% (n = 14) of hip shimmy attempts, by initiating additional gestures or gesture sequences that repeated (26.7%, n = 12) or elaborated (73.3%, n = 33) upon the initial gesture. Elaborations included non-referential gestures (n = 26), foot-pointing or hip shimmy gestures (n = 3), or tactile gestures (n = 7, see Supplementary Fig. 1 and Supplementary Table 1). When females used persistence, the majority of these gesture bouts (75.6%, n = 34) resulted in GG rubbing (Fig. 2).

Of the unsuccessful gesture bouts, in most cases (78.3%, n = 18) the intended recipient ignored the signaller. In the remaining five unsuccessful bouts, recipients responded more strongly by turning away (n = 3), moving away (n = 1), or aggressing against the signaller (n = 1). The initial positive or negative response to gestural solicitations also influenced females’ subsequent behaviour. Of the gestural solicitations that occurred in feeding contexts (n = 135), signallers and recipients were more likely to co-feed within close proximity on the same resource following positive (66.1%, n = 74) compared with negative (34.8%, n = 8) responses to gestural solicitations (GLMM, estimate ± SE = 1.30 ± 0.489, *P* = 0.008, see Supplementary Table 3).

## Discussion

These results provide evidence that wild female bonobos at Luikotale use a referential gesture and a potentially iconic gesture to communicate their intention to engage in GG rubbing with other females and to induce behavioural coordination with recipients. Moreover, GG rubbing occurs at greatly reduced rates in the same social feeding contexts but without the use of these gestures, indicating that GG rubbing is not merely a general response to social feeding. In the foot-pointing gesture, the directed movement of the signaller’s heel or toes functions as a location indicator and the signaller’s sexual swelling serves as a visual referent for the act of GG rubbing. In the hip shimmy, the praxic action of the signaller pantomimes the rapid lateral movement of the hips that occurs during GG rubbing. The iconic nature of this gesture is further suggested by the observation that females use the hip shimmy exclusively when soliciting GG rubbing, and not in other contexts where the action is not relevant, for example when soliciting copulations with males.

Foot-pointing and hip shimmies performed as single gestures were highly successful in eliciting a specific response that incorporated the referenced location or the putative desired action. This indicates that both gestures have a strong communicative function. The encoded information was understood by other females in the community as well as by recent female immigrants from other communities. In the minority of cases when a recipient responded in an undesired way to a single foot-point or hip shimmy, females showed persistence by repeating or elaborating upon their initial gesture, consistent with data from studies of communicative persistence in captive apes^8^,^31^,^37^ and wild chimpanzees^38^. This demonstrates flexibility by the signaller in attaining her goal.

Although the signaller sometimes made direct contact with her sexual swelling during the pointing, the context in which the gestures occurred contrasts with anecdotal evidence of masturbation in wild and captive female bonobos, which typically occurs in asocial contexts and is not followed by socio-sexual interactions with other individuals. Moreover, our analysis showed that receivers responded to pointing gestures in the same way whether or not the signaller made contact with her swelling, suggesting that pointing with or without genital contact serves a similar communicative function.

It is important to acknowledge that while we refer to these gestures as forms of pointing and pantomime, we cannot be certain that their production and comprehension involve the same underlying cognitive processes as occurs in referential and iconic communication between humans. For example, we cannot determine whether females use the hip shimmy gesture because they are aware that the action performed physically resembles the putative goal of GG rubbing. This is a necessary criterion for pantomime as used in human communication^39^. Similarly, we cannot determine whether the recipients respond to the hip shimmy because they recognise that the signaller is intentionally imitating the act of GG rubbing, as a means of initiating it, or because they simply associate the observed act with the act of GG rubbing. Additionally, we do not make any claims about the ontogeny of these gestures. There are parallels between the hip shimmy described here and the “hip shaking” gesture reported by Rossano^40^, produced by a female bonobo in captivity to invite her infant to engage in a ventral carry. Rossano suggested that the hip shaking gesture likely emerged through ontogenetic ritualisation in this group^40^. One possibility is that the hip shimmy is also ontogenetically ritualised from familiar action sequences. Were this true then, while the hip shimmy looks iconic in appearance, bonobos would be able to use the gesture communicatively without the gesture qualifying as iconic. Nonetheless, the recording of this gesture is important because, like the beckoning gesture previously observed among bonobos^21^, it constitutes a promising candidate for a gesture that may be being used iconically. The clear evidence of referentiality that is provided by the foot-pointing gesture, together with the potentially iconic nature of the hip shimmy, provide compelling evidence that wild, non-enculturated apes communicate using language precursors that have been implicated in the evolution of theory of mind and human language^3^,^9^.

Recent studies have emphasised the potential role of environmental and social conditions, along with evolved cognitive capacities, in explaining inter-specific variation in the use of referential gestural communication^26^,^37^. For example, new evidence that two species of fish use pointing-like gestures to recruit heterospecific helpers for collaborative hunting highlights the role of ecological necessity in the emergence of referential gestures^26^. If referential gesturing emerges in part to overcome environmental barriers, this may also explain the contrasting evidence that apes demonstrate the ability to point in captivity^12^,^41^,^42^, but show little evidence for pointing or other types of referential gesturing in the wild. Physical barriers typically present in captivity, for example in accessing out of reach objects or foods, may create a functional need for these gestures^43^.

Evidence from captive and field settings suggests that GG rubbing can reduce social tension and facilitate cooperation among female bonobos, especially in potentially competitive contexts involving monopolisable foods^44^,^45^. In addition, females whose socio-sexual solicitations are rejected may suffer social costs, such as reductions in social status^34^,^46^. If referential and iconic gestures increase the likelihood of GG rubbing, and if such behavioural coordination with other females helps to overcome social barriers in gaining access to resources, then females should use these gestural solicitations more frequently in situations where they face such barriers. Overall, 18.7% of feeding minutes during which foot-pointing or hip shimmies occurred involved feeding on highly monopolisable foods (fruits of *Annonidium* or *Treculia* species, or meat), while feeding on the same monopolisable foods accounted for only 4.3% of feeding minutes when referential or iconic gestures were not used. This suggests that referential and iconic gestures are associated with potentially competitive feeding contexts in which high levels of social tolerance and cooperation are required to share resources. Signallers were also more likely to co-feed within close proximity to recipients following successful versus unsuccessful gestural solicitations. This further demonstrates the link between these gestures, GG rubbing, and the facilitation of cooperative behaviours. That female bonobos were only observed to use foot-pointing and hip shimmies in interactions with other females suggests that intra-sexual behavioural coordination may play a more prominent role than inter-sexual coordination in overcoming social barriers, a suggestion that is consistent with other studies^45^,^47^,^48^.

Based on a review of the literature and the results of this study, we suggest that the following conditions may facilitate the use of referential or iconic gestural communication: (i) presence of ecological or social barriers in gaining access to preferred resources, (ii) the necessity for behavioural coordination and cooperation with others to overcome these barriers, (iii) being a member of the dispersing sex, in which barriers must be overcome without the help of relatives, and (iv) a social system characterised by relatively low levels of overt aggression and a less steep dominance hierarchy, in which there are many possible partners for cooperation. These conditions are all applicable for female bonobos, who appear to use GG rubbing to diffuse social tension and facilitate cooperation specifically among unrelated females, providing a range of fitness benefits including female-biased possession and sharing of preferred, monopolisable foods^45^,^48^.

While the majority of mature female residents of the Bompusa community use one or both of these gestures, we observed high inter-individual variation in the frequency of foot-pointing and hip shimmies, and in female preferences for the use of different gestures. This variation was not related to differences in observation time, suggesting that females differ in their reliance on specific types of gestures when communicating with others. Such variation may be related to individual differences in social status or social integration, which in turn may affect the various strategies that females use to elicit GG rubbing with others^46^,^49^.

In addition to the referential and potentially iconic gestures we have described here, females from the Bompusa community also use other intentional gestures in socio-sexual solicitations (see Supplementary Table 1). These gestures are used more broadly in inter- as well as intra-sexual solicitations and therefore can result in different socio-sexual interactions including, but not limited to, GG rubbing. Future research should determine whether referential and iconic gestures are more effective than other intentional gestures in eliciting GG rubbing, and identify conditions that promote differential use of referential and iconic gestures versus other gestures among these female bonobos.

This study reveals that two gestures resembling pointing and pantomime in form and function are constituents in the communicative repertoire of wild female bonobos. By directing attention to a specific referent or action involved in the desired goal, these gestures are used regularly to facilitate behavioural coordination with female conspecifics. The capacity for referential and iconic gesturing demonstrated in this community of wild bonobos substantiates the argument that nonhuman primates use language precursors to enhance communication in contexts in which behavioural coordination and cooperation are necessary.

## Methods

### Subjects and data collection

Between January 2011 and June 2014, we observed the Bompusa community of habituated wild bonobos at the Luikotale field site, Democratic Republic of Congo^50^. Bonobos live in large, multimale multifemale communities characterised by a high degree of fission-fusion social dynamics, referring to the fluid splitting and merging of individuals in subgroups or parties that fluctuate in size and composition^51^. During the observation period, the community consisted of n = 13–14 mature females (≥10 years), n = 6–7 mature males, and n = 11–20 immature individuals. All gesturing and GG rubbing events were recorded by P.H.D., L.R.M., and one trained research assistant during 2,740 contact hours using focal-animal and all occurrence sampling^52^. Gestures were coded in real time (n = 134) or, when possible, were captured using HD video cameras (Panasonic HC-V700M and Sony HDR-PJ380) and analysed later (n = 35).

### Definitions and operational criteria

In accordance with the primate literature^37^,^53^,^54^, intentional gestures were defined as discrete movements of the limbs or body that were directed towards a recipient, adjusted to the attentional state of the recipient, mechanically ineffective, goal-directed, and were followed by persistence and/or elaboration of the gesture when initial communicative attempts were not successful. Among several intentional gestures that we observed frequently in the context of socio-sexual solicitations (see Supplementary Table 1), two types of gestures, termed “foot-pointing” and “hip shimmy”, were of special interest because they appeared to incorporate additional referential or iconic information about the desired outcome, specifically GG rubbing with the recipient of the gesture. The foot-pointing gesture was characterised by the rapid, directional movement of one lower limb and foot towards the signaller’s sexual swelling, with or without actual contact with the sexual swelling, and with or without repetition (see Supplementary Movie 1). The hip shimmy gesture was defined as rapid lateral movement of the hips in a ventral present position, usually achieved by hanging with both arms from a substrate (see Supplementary Movie 2).

A *single gesture* was defined as one foot-pointing or hip shimmy gesture, followed by a pause of >1 s^40^,^55^. A *gesture sequence* contained at least one referential or iconic gesture, in combination with other referential, iconic, and/or intentional gestures, separated by ≤1 s. *Gesture bouts* consisted of multiple single gestures or gesture sequences separated by a pause of between 1 and 30 s, and performed by the same individual in the same context. When foot-pointing and hip shimmies were combined in one gesture bout, the last gesture of the sequence was used to categorise gesture bouts for analyses, following Halina and colleagues^56^. We recorded any changes in the recipient’s behaviour occurring within 60 s following the end of a gesture bout. A positive response was scored when the recipient engaged in GG rubbing with the signaller within 60 s following the end of the gesture bout. Neutral or negative responses included ignoring the signaller (no change in behaviour), turning away from, moving away from, or aggressing against the signaller. If the signaller initiated another gesture during or following the 60 s response period, this was considered an independent gesture bout.

*Communicative persistence* was defined as repetition or elaboration of a single gesture or gesture sequence when it did not elicit the desired response^37^,^38^. *Elaboration* could include the addition of a different referential or iconic gesture, or the addition of other intentional gestures including ventral present, head shake, touch, and body rock (see Supplementary Table 1 and Supplementary Figure 1). When signallers showed persistence, we scored recipients’ responses until the goal was achieved or until the recipient clearly indicated a negative response.

### Behavioural coding

The following variables were coded for each gesture bout: identity of signaller and recipient; gesture type (determined by the last referential or iconic gesture in the gesture bout); behavioural context (feeding, aggression, playing, resting, or travelling); stable or changing social context (defined as occurrence of the gesture within 5 min following a fission or fusion event, or after one or both interactants entered a new food patch); attentional state of the recipient; and whether the signaller or recipient approached within close proximity (defined as ≤2 m) before or during the gestural solicitation. The attentional state of the recipient was inferred from the recipient’s body direction, and was coded relative to the signaller as: facing (0–45°), within the visual range of the signaller (45–90°), or outside the signaller’s visual range (>90°). Following the gesture, we recorded the recipient’s initial behavioural response as positive (GG rubbing) or negative (ignore, turn away, move away, aggress). In addition, we recorded the proximity and behaviour of both participants following the recipient’s initial response. When participants remained within close proximity and both fed, this was termed co-feeding and was considered a measure of social tolerance.

### Analyses

P.H.D., L.R.M., and one trained assistant coded a subset of foot-pointing and hip shimmies together in the field. P.H.D. and L.R.M. further assessed inter-observer reliability by independently coding a random sample of fifteen video segments. Cohen’s Kappa values indicated a good to perfect level of agreement between the coders in: identifying every gesture (foot-pointing, hip shimmy, other intentional gestures) within a bout (K = 0.636), identifying only referential or iconic gestures within a bout (K = 1.0), determining the attentional state of the recipient (K = 0.765), identifying the recipient’s response (K = 1.0), and determining whether persistence occurred (K = 1.0). We described patterns of gesturing and the socio-ecological contexts in which gestures occurred for all independent gesture bouts observed (n = 169). We further analysed only the subset of gesture bouts from gestures coded in real time or captured on video, that occurred in clearly social contexts and where all criteria for intentionality, referentiality, and iconicity could be assessed (n = 138). Statistical analyses were performed in R 3.1.2^57^ using non-parametric Spearman and Wilcoxon tests. To determine whether the use of foot-pointing and hip shimmies increased the likelihood of GG rubbing, we calculated each female’s baseline rate of GG rubbing while feeding on non-monopolisable foods in social contexts, when referential or iconic gestures were not used. We then compared this baseline rate with the female’s rate of GG rubbing while feeding on the same non-monopolisable food types, but when she used referential or iconic gestural solicitations. In both conditions, we only analysed social feeding contexts in which two or more females were present in the same feeding patch. Only feeding contexts involving non-monopolisable foods were analysed, since feeding on monopolisable foods is associated with increased GG rubbing interactions^44^,^45^. Results are presented as 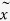 (range).

We also ran two Generalized linear mixed models (GLMMs) with the package lme4^58^. The first model tested whether foot-pointing gestures in which the female’s foot made contact with her swelling (test predictor) were less likely to result in GG rubbing (response variable). If contact with her swelling served a different function, such as self-stimulation, then it might be considered a mechanically effective action instead of a gesture to another individual. The second model tested whether a recipient’s acceptance or rejection of a gestural solicitation (test predictor) influenced the likelihood of co-feeding following a gestural solicitation (response variable). For this model, we included all gesture bouts that occurred in social feeding contexts (n = 135). In both models the identity of the signallers, recipients, and dyads were included as random effects. In addition, we included random slopes of each test predictor within individuals and dyads in both models to reduce the possibility of inflated type I error rates^59^. We used likelihood ratio tests to compare each full model to a null model excluding the test predictor, and present results of models that differed significantly from the null model.

## Additional Information

**How to cite this article**: Douglas, P. H. and Moscovice, L. R. Pointing and pantomime in wild apes? Female bonobos use referential and iconic gestures to request genito-genital rubbing. *Sci. Rep.* **5**, 13999; doi: 10.1038/srep13999 (2015).

## Supplementary Material

Supplementary Information

Supplementary Movie 1

Supplementary Movie 2

## Figures and Tables

**Table 1:** Details of signallers and gesture bouts produced by each female.

**Signaller**	**Age class**	**Number of pointing bouts**	**Number of hip shimmy bouts**	**Percentage of produced gesture bouts that led to GG rubbing**	**Total observation time per female (hrs)**
Dj	Adolescent	19		73.7	494.0
Gw	Adult	55		76.4	799.5
Ir	Adult	17		94.1	1176.5
Lu	Adolescent	2		100	818.0
Ma	Adult	11	27	94.7	1061.5
Ol	Adult	9	1	70.0	1252.0
Pa	Adult		1	100	1099.0
Po	Adolescent	7	3	70.0	1168.0
Su	Adult	8	2	100	1237.5
Wi	Adult	1		100	941.0
Zo	Adult	1		100	1430.0
		Total 130	Total 34		

**Table 2:** Details of recipients and responses to gesture bouts.

**Receiver**	**Age class**	**Number of pointing bouts**	**Number of hip shimmy bouts**	**Percentage of received gesture bouts that led to GG rubbing**	**Receiver also a signaller**	**Tenure in community**
Dj	Adolescent	6	6	100	Yes	Since 2012
Gw	Adult	1	1	100	Yes	Since 2002
Ir	Adult	8	2	40.0	Yes	Since 2002
Lu	Adolescent	4		50.0	Yes	Since 2008
Ma	Adult	9		77.8	Yes	Since 2002
Ol	Adult	8	1	88.9	Yes	Since 2002
Pa	Adult	5		60.0	Yes	Since 2002
Po	Adolescent	5	6	90.9	Yes	Since 2002
Su	Adult	15	2	88.2	Yes	Since 2002
Wi	Adult	7		85.7	Yes	Since 2009
Zo	Adult	12	6	94.4	Yes	Since 2002
Ag*	Adult	1		0.0	No	Temporary
Fy	Adolescent	2	2	100	No	Temporary
Na	Adult	17	1	83.3	No	Since 2010
Ng	Adolescent		1	100	No	Temporary
Op	Juvenile	3	1	100	No	Since 2009
Pg	Juvenile	3		100	No	Since 2006
Ri	Adult	10	5	93.3	No	Since 2002
So	Juvenile	2		100	No	Since 2009
Um	Adult	9		66.7	No	Since 2002
Ve	Adolescent	1		0.0	No	Temporary
Vl	Adolescent	1		100	No	Temporary
Wa	Juvenile	1		100	No	Since 2011

Organised alphabetically and by whether the receiver was also a signaller.

*This female, from the East Community, was gestured to during an intercommunity encounter.
