# Supplementary material for: Pointing and pantomime in wild apes? Female bonobos use referential and iconic gestures to request genito-genital rubbing
Source: Sci Rep. 2015 Sep 11;5:13999. doi: 10.1038/srep13999 (PMC4642555; doi:10.1038/srep13999)
Supplement: Supplementary Information [file srep13999-s1.pdf]

Pointing and pantomime in wild apes? Female bonobos use referential and iconic gestures to request genito-genital rubbing

Pamela Heidi Douglas & Liza R. Moscovice

Supplementary Information

Supplementary Table 1

Table 1. Ethogram describing other intentional gestures used by females to solicit GG rubbing interactions.

Gesture	Description
Bipedal stand	Stand bipedally on both legs, facing recipient with an upright posture
Head shake	Rapidly nod head up and down
Ventral present	Spread legs open to present genital region ventrally while facing a recipient
Rock	Rock torso with a back to front motion
Touch	Gently touch or nudge recipient, usually using a foot

Supplementary Table 2. Comparison of GG rubbing rates per minute, without and with referential or iconic gesturing, while feeding on non-monopolisable foods.

Signaller	GG rate without a referential or iconic gesture	GG rate following a referential or iconic gesture
Dj	0.006	0.778
Gw	0.002	0.719
Ir	0.002	1.000
Lu	0.004	1.000
Ma	0.003	0.964
Ol	0.002	0.571
Po	0.004	0.571
Su	0.005	1.000
Wi	0.004	1.000
Zo	0.003	1.000

Supplementary Table 3. Results of a GLMM comparing the likelihood of co-feeding following successful or unsuccessful gestural solicitations.

Significant predictors are indicated in bold typeface.

Response Variable	Estimate	SE	*P value*
Co-feed following gesture (yes/no)			
Intercept	-0.6286	0.4378	
**Gesture leads to GG rubbing**	**1.3072**	**0.4889**	**0.0075**

Supplementary Figure Legend

Supplementary Figure 1. Description of elements within gesture bouts. The relative amount and position of different gesture types within gesture bouts, including foot-pointing, hip shimmies, and other intentional gestures. Shading indicates different gesture types. Intentional gestures include bipedal stand, head shake, ventral present and rock. Numbers over the bars indicate the total number of gesture bouts of each length (shown on the x-axis).

Supplementary Figure 1

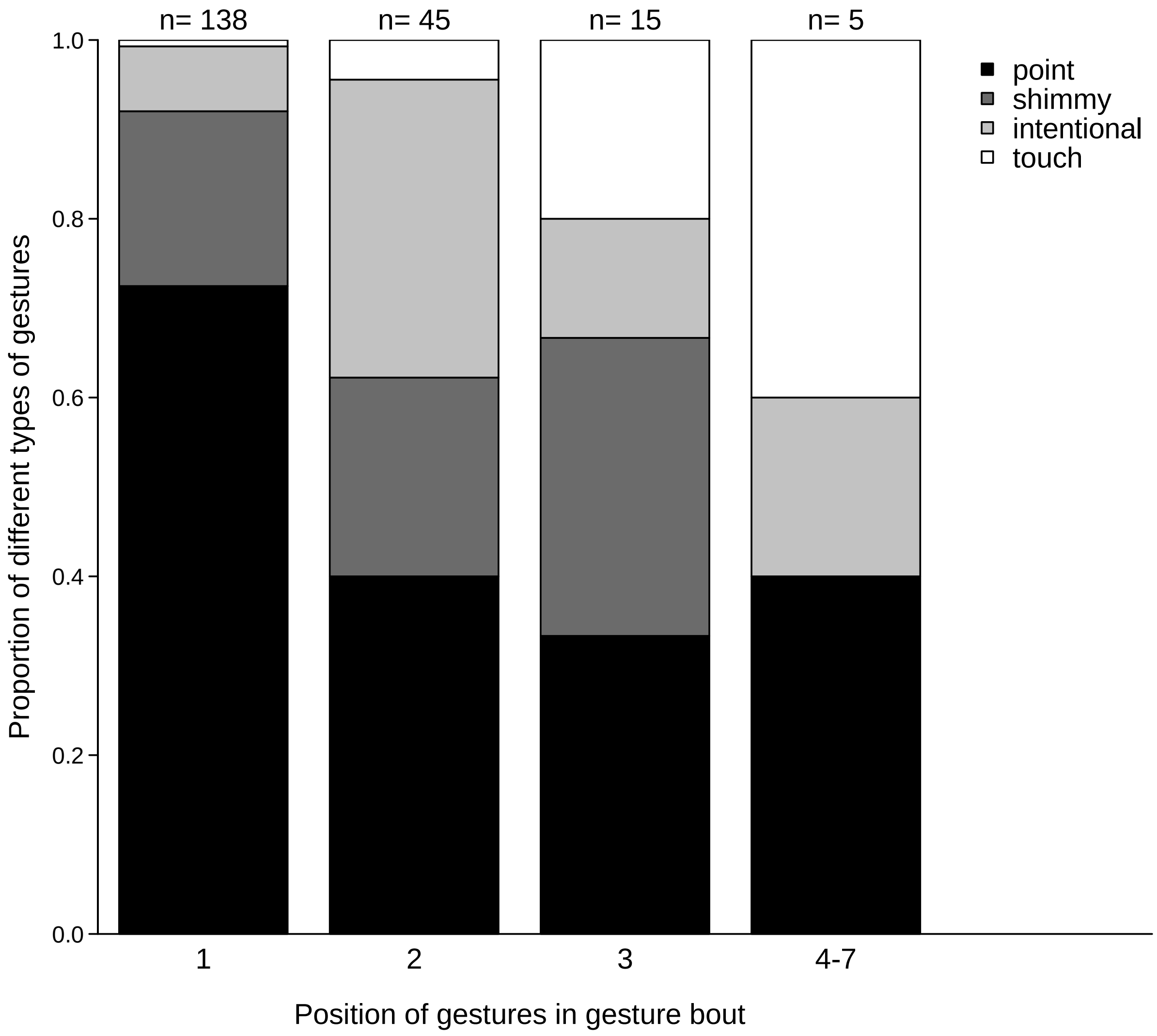

Supplementary Movies

Supplementary Movie 1. Example of foot-pointing gesture.

At 00:02, Dj is initiating a solicitation to Na who is feeding. A yellow circle surrounds Dj's right foot during her pointing gesture, and the footage has been slowed down during the pointing to assist in detecting the gesture. The females then engage in prolonged GG rubbing. Afterwards, the females co-feed.

Supplementary Movie 2. Example of hip shimmy gesture.

At 00:00, Ma is soliciting to Ri who is feeding (initially outside the frame). A yellow circle surrounds Ma's hips and sexual swelling during her hip shimmy. Ma moves closer to Ri, and then shows persistence in gesturing again with a ventral present and second hip shimmy at 00:19 (partly obstructed by foliage). The females then engage in a GG rubbing.
